# Serological investigation on *Sarcocystis* spp. infection and associated risk factors in South American camelids in Italy

**DOI:** 10.1007/s00436-026-08655-9

**Published:** 2026-03-04

**Authors:** Elisa Castaldo, Gastón Moré, Gereon Schares, Francesco Buono, Stefano Scarcelli, Giovanni Sgroi, Farwa Humak, Walter Basso, Vincenzo Veneziano

**Affiliations:** 1 Department of Veterinary Medicine and Animal Productions, University of Naples Federico II, Naples, Italy; 2 Institute of Parasitology, Vetsuisse-Faculty, University of Bern, Bern, Switzerland; 3 Federal Research Institute for Animal Health, Institute of Epidemiology, Friedrich-Loeffler Institut, Greifswald-Insel Riems, Germany; 4 Department of Animal Health, Experimental Zooprohylactic Institute of southern Italy, Portici, Italy; 5 Department of Sciences and Technologies, University of Sannio, Benevento, Italy

**Keywords:** Aalpacas, IFAT, Italy, llamas, *Sarcocystis*, Seroprevalence

## Abstract

Protozoa of the genus *Sarcocystis* have an obligate two-host-prey-predator life cycle, in which South American Camelids (SAC) are among the intermediate hosts for certain species. *Sarcocystis* spp. infection has been well documented in SAC in endemic areas of the South America, while data from Europe are absent. This study assessed the seroprevalence of *Sarcocystis* spp. in SAC in Italy investigating related risk factors. A total of 506 SAC sera (486 alpacas and 20 llamas) from 38 sampling sites were analysed to detect antibodies to *Sarcocystis* spp. using the Indirect Fluorescent Antibody Test (IFAT). A seropositivity of 15.2% (77/506) was found, corresponding to 14.4% (70/486) in alpacas and 35.0% in llamas (7/20). Risk factor analysis showed that *Sarcocystis* spp. seropositivity increased with age, while no statistically significant association was found with regard to sex. Access to stables and/or pasture by dogs was identified as a putative risk factor, particularly as the number of dogs increased. Further association was found between seropositivity to *Sarcocystis* spp. and *N. caninum*. Although the life cycles of *Sarcocystis* spp. in SAC are not fully elucidated, these findings highlight the need for further studies to clarify the potential role of dogs as definitive hosts for *Sarcocystis* species infecting SAC in Europe. This study provides for the first time data on specific antibodies for *Sarcocystis* spp. in SAC in Europe, highlighting the need of improving the surveillance of this protozoan in camelids, particularly given the growing interest in camelid meat production.

**Supplementary Information:**

The online version contains supplementary material available at https://doi.org/10.1007/s00436-026-08655-9.

## Introduction

Protozoa of the genus *Sarcocystis* (Phylum Apicomplexa) have an obligatory prey-predator life cycle, and include over 200 species infecting birds, reptiles, fish, and mammals, including humans as either the definitive or the intermediate host (Dubey et al. 2016). Definitive hosts (carnivores and/or omnivores) acquire the infection by ingesting mature sarcocysts containing bradyzoites which penetrate the mucosa of the small intestine, develop into gamonts and initiate the sexual development (gametogony), leading to oocysts formation. The oocysts sporulate in the intestine (sporogony), and free sporocysts are excreted through the faeces. Intermediate hosts (prey animals) get infected by ingesting sporocysts while grazing on pasture or through contaminated water. Sporocysts release sporozoites, which undergo asexual development (schizogony) with merozoites formation and binary division (endodyogeny). Depending on *Sarcocystis* species, invasion of the appropriate host cells (mainly cardiac and/or skeletal muscle) by merozoites leads to the formation of sarcocysts containing bradyzoites (Dubey et al. 2016).

Camelids can serve as intermediate hosts and different *Sarcocystis* spp. have been described in Old World Camelids (OWC), as well as in New World Camelids (NWC) (Wieser et al. 2024). The group of OWC includes the dromedary (*Camelus dromedarius*) and the Bactrian camel (*Camelus bactrianus*), indigenous to Africa and Asia. Whereas, NWC include domestic species, such as alpacas (*Vicugna pacos*) and llamas (*Lama glama*), along with wild species like vicuñas (*Vicugna vicugna*) and guanacos (*Lama guanicoe*). Collectively, NWC are also called South American Camelids (SAC) due to their geographic origin (Fowler 2010). The taxonomic classification of *Sarcocystis* species infecting camelids has long been complex and debated. However, critical and thorough re-evaluations have led to the validation of four species: *S. cameli* and *S. ippeni* in OWCs, and *S. aucheniae* and *S. masoni* in SAC (Dubey et al. 2015, 2016; Moré et al. 2016). The life cycle of these species has not been fully elucidated. Although dogs are recognized as experimental competent definitive hosts of these species, the role of canids as natural definitive hosts needs to be clarified (Wieser et al. 2024).

Diagnosis of sarcocystosis is mainly based on gross examination of carcasses during necropsy, along with histopathological examination (Wieser et al. 2024). However serological (Moré et al. 2008a; Romero et al. 2017) and molecular techniques (Decker Franco et al. 2018) have also been developed for in live diagnosis.

Infection with *Sarcocystis* spp. is generally subclinical in SAC, however cases of eosinophilic myositis and abortion have been reported in alpacas (La Perle et al. 1999; Gabor et al. 2010). In humans, the consumption of undercooked meat could lead to gastroenteritis, chills, nausea, diarrhoea, colic, and respiratory illness due to the presence of a peptidic enterotoxin – sarcocystin - in the cysts (Saeed et al. 2018; Wieser et al. 2023). Although evidence for the zoonotic potential of SAC sarcocystosis is still lacking, the presence of *S. aucheniae* macroscopic sarcocysts in meat, resembling “rice grains”, reduce commercial value, leading to considerable economic losses (Romero et al. 2017; Miranda-de la Lama et al. 2022).

Historically, the consumption of domesticated SAC meat has been limited to the Andean countries, where most studies on sarcocystosis in these animals have been carried out, reporting prevalences up to 100% (Fernandez et al. 2022). However, over the past 15 years, demand for this meat has increased also from North America, Oceania, and Europe (Miranda-de la Lama et al. 2022). In Italy, SAC meat is not currently commercialized (Castaldo et al. 2025); nevertheless, due to its high quality, sensory and nutritional parameters (Popova et al. 2021) a commercialization of SAC meat in Italy, similar to other European countries, cannot be ruled out.

This study determined the seroprevalence of *Sarcocystis* spp. infection in alpacas and llamas in Italy, investigating the risk factors associated with the presence of *Sarcocystis* spp. specific antibodies.

## Materials and methods

### Study animals and sampling sites

During routine veterinary clinical examinations, blood samples were taken from the jugular vein of 506 SAC (i.e., 486 alpacas and 20 llamas) and placed in 10 ml tubes without anticoagulant (APTACA^®^, Italy). Samples were refrigerated, then delivered to the Department of Veterinary Medicine and Animal Productions of the University of Naples “Federico II” where sera were separated after centrifugation (3000 x *g*, 10 min) and stored at − 20 °C until serological analysis performed at the Institute of Parasitology, Vetsuisse Faculty, Bern University (IPB). The same sera had previously been tested for the detection of *Toxoplasma gondii* and *Neospora caninum* antibodies by ELISA as reported by (Castaldo et al. 2025).

During sampling, animal identification number, sex, and age were recorded from each animal.

Of the study animals, 64.2% (325/506) were females, 31.6% (160/506) males, and 4.2% (21/506) castrated. The animals were classified into four age-classes as crias (≤ 6 months), weaners (> 6 months - < 12 months), tuis (≥ 1 year - < 2 years), and adults (≥ 2 years) (Love 2017). Accordingly, 4.2% (21/506) were crias, 4.0% (20/506) weaners, 12.8% (65/506) tuis, and 79.1% (400/506) were adults. The mean age of animals ± standard deviation was 4.8 ± 3.9 years (range: 1 month – 23 years).

**Fig. 1:**
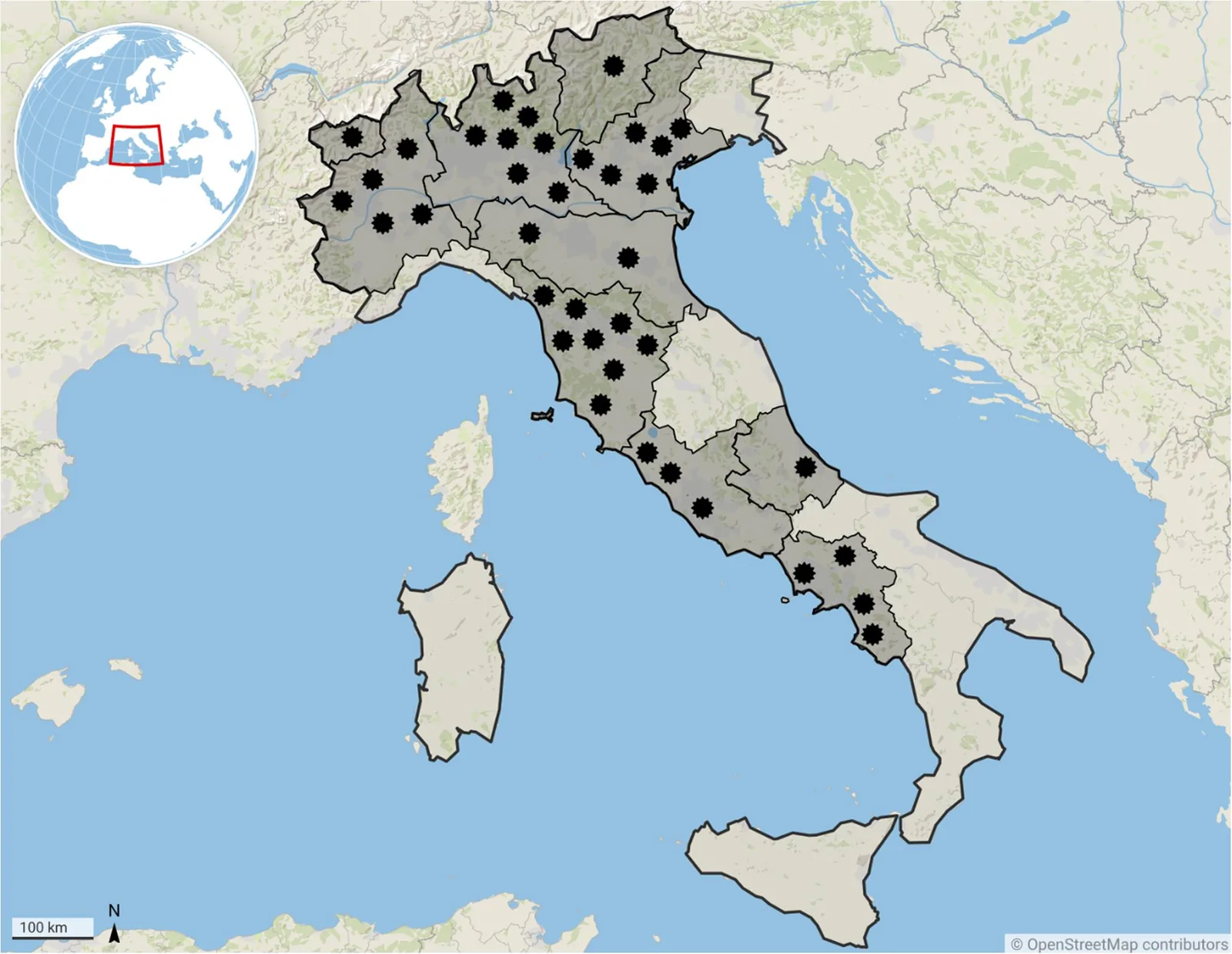
Distribution of sampling sites indicated by stars (*n* = 38) where the presence of antibodies against *Sarcosystis* spp. was assessed

The animals were sampled from 38 sampling sites located in northern, central, and southern Italy. In detail, out of 38 sampling sites (i.e., 24 farms, 8 zoos, and 6 private gardens in which SAC were kept as companion animals), 57.9% (22/38) were located in northern Italy, including a total of 200 animals (39.5%), 31.6% (12/38) in central Italy with a total of 281 animals (55.5%), and 10.5% (4/38) in southern Italy with a total of 25 animals (4.9%) (Fig. 1).

### Questionnaire

To identify possible risk factors for *Sarcocystis* spp. infection, the owners of each sampling site filled an online questionnaire providing information on sampling site structure, management practices, sanitary controls and clinical history of the animals (Supplementary material S1).

### Indirect Fluorescent Antibody Test (IFAT)

The presence of antibodies to *Sarcocystis* spp. in SAC sera was determined by the Indirect Fluorescent Antibody Test (IFAT) in a similar way as previously described by (Moré et al. 2008a, b). Although sensitivity and specificity data for SAC are not available, this method has been widely used for the detection of genus-specific antibodies in living animals and is considered highly sensitive (Dubey et al. 2016). Briefly, muscle samples (100 g) from naturally infected wild boars with *S. miescheriana* were homogenized, then mixed with digestion solution (400 ml), incubated, centrifuged and filtrated (Moré et al. 2008b, 2025). Free bradyzoites in a final concentration of 4 × 10^6^ bradyzoites/ml were transferred to 12-well IFAT slides, allowed to dry, and freeze at – 20 °C for at least 24 hours. Wells were incubated with sera (diluted 1:25 in Phosphate Buffered Saline **-** PBS) in a humid chamber, washed and incubated with goat anti-llama IgG (Cat. No.: 6045-02, Southern Biotech, USA) fluorescein isothiocyanate conjugate (diluted 1:100 in PBS), followed by washing. The slides were mounted with mounting fluid (glycerol/PBS) and a cover slide, then observed by at least two trained operators (E.C., G.M., W.B.) in an epifluorescence microscope (Leitz Laborlux S, LED light) at 400 x magnification. Complete peripheral fluorescence of bradyzoites was considered as positive result, and positive samples at 1:25 were then re-tested at 1:50 and 1:100 dilutions. Bovine and alpaca sera from naturally infected animals with *S. cruzi* and *Sarcocystis* spp., respectively, were used as positive controls. Sera from alpaca with no detectable reaction in the first screening, and PBS were used as negative controls.

### Statistical analysis

To identify risk factors associated with *Sarcocystis* spp. seropositivity, a binomial Generalised linear mixed-effect models was used, incorporating the information on sampling sites and individual animals. All potential predictor variables were derived from the questionnaire administered to SAC owners. Based on IFAT results, the animal serostatus (i.e., seropositive or seronegative) for *Sarcocystis* spp. was included as the binomial dependent variable, while the potential risk factors were considered as predictor variables. Given that individual animal clustered in sampling sites, the variable sampling site ID was considered as a random effect variable. Each variable was tested in association with the serostatus of individual animals for the infection. Multilevel-modelling at animal level was performed using function *glmer* (package “lme4” with Laplace method as default approximation), binomial distribution, binary response variable (i.e., animal serostatus) with values (i.e., seropositive or seronegative), random factor (i.e., sampling site ID), and effect modifier (i.e., age in years). Variables that showed a statistically significant association (*p <* 0.05) in the bivariable models (including in addition to the explanatory variable also age) were selected for inclusion in the multivariable model. Variables identified as potential confounders were excluded from the multivariable model. The null hypothesis (H0) aimed to prove the independence between the infection status and the corresponding risk factors (categorical variables). Statistical modelling was made in R 4.4.2 (http://www.R-project.org). The Akaike Information Criterion (AIC) was used to compare the relative support for competing models; the lower the AIC the better a model performs relative to the other models presented. Data included in the statistical analysis were compared with the results obtained in the previous study on *T. gondii* and *N. caninum* to evaluate possible cross-reactions.

## Results

### Questionnaire

Filled questionnaires were obtained from the owners of all sampling sites (100.0% − 38/38). Most respondents managed small herds (≤ 20 animals) (76.3%, 29/38), followed by medium (21–50 animals) (15.8%, 6/38) and large (> 50 animals) (7.9%, 3/38). The main purposes of keeping animals were fibre production (43.0%, 16/38), while none kept animals for meat production. Among the owners, 55.3% (21/38) reported keeping Italian SAC (born at the same farm or purchased from other Italian farms), whereas 44.7% (17/38) also kept animals originating from abroad, like other European countries (i.e., Germany 52.9%, Switzerland 41.2%, Belgium 17.6%, France 17.6%, United Kingdom 17.6%, Austria 5.9%, and the Netherlands 5.9%) or Oceania (i.e., New Zealand 11.8% and Australia 5.9%), South America (i.e., Chile 11.8%, and Peru 5.9%) and North America (i.e., USA 5.9%). Owners could report multiple countries of origin. Animals had access to pasture in almost all sampling sites (81.6% − 31/38). More than half (52.6%, 20/38) of the respondents also bred domestic ruminants. Regarding domestic pets, 23.7% (9/38) of the respondents owned cats at sampling time, and 42.1% (16/38) reported having housed cats in the last two years. In 52.6% (20/38) of the sampling sites, both owned cats and non-owned cats (i.e., stray cats or neighbour’s cats) were reported to have access to the stables and/or pasture where SAC were held. Regarding dogs, 55.3% (21/38) reported the presence of owned dogs at sampling time, and 68.4% (26/38) have had dogs in the last two years. Dogs (both owned and non-owned) had access to the stables and/or pasture in 52.6% (20/38) of the sampling sites.

### Indirect Fluorescent Antibody Test (IFAT)

Antibodies to *Sarcocystis* spp. (IFAT titre ≥ 25) were found in 15.2% (77/506, 95% CI 12.1–18.3) of serum samples. In detail, a seroprevalence of 14.4% (70/486, 95% CI 11.3–17.5) was found in alpacas and a seropositivity of 35.0% (7/20, 95% CI 14.1–55.9) in llamas. Of the positive samples, 9.1% (7/77, 95% CI 2.7–15.5) also tested positive at titre 50, and 1.3% (1/77, 95% CI 0–3.8) at titre 100. The seroprevalence at the sampling site level was 55.3% (21/38, 95% CI 39.5–71.1).

Regarding sex, seropositivity was 16.9% (55/325, 95% CI 12.8–21) in females, 10.6% (17/160, 95% CI 5.9–15.4) in males, and 23.8% (5/21, 95% CI 5.6–42.0) in castrated animals.

With respect to age-classes, no seropositive crias were found, while 10.0% (2/20, 95% CI 0–23.1) of weaners, 7.7% (5/65, 95% CI 1.2–14.2) of tuis, and 17.5% (70/400, 95% CI 13.8–21.2) of adults tested seropositive to *Sarcocystis* spp.

Additional data on the seroprevalence for *Sarcocystis* spp. are reported in Table 1.

**Table 1:** Seropositivity for *Sarcocystis* spp. at animal (*n* = 506) and sampling site (*n* = 38) levels according to the main variables included in the study

Variable	Animals		Sampling sites	
	Positive (%, *n*)	95% CI	Positive (%, *n*)	95% CI
Area				
Northern	14.5% (29/200)	9.62–19.38	59.1% (13/22)	38.55–76.94
Central	17.1% (48/281)	12.68–21.48	66.7% (8/12)	39.99–93.34
Southern	0.0% (0/25)	0	0.0% (0/4)	0
Herd size				
Small	17.5% (32/183)	11.98–22.99	55.2% (16/29)	37.07–73.27
Medium	10.8% (10/93)	4.46–17.05	50.0% (3/6)	9.99–90.01
Large	15.2% (35/230)	10.58–19.86	66.7% (2/3)	13.32–100.00
Type of housing				
Stable with access to paddock	13.9% (14/101)	7.12–20.60	40.0% (6/15)	15.21–64.79
Stable with access to pasture	15.6% (63/405)	12.03–19.09	65.2% (15/23)	45.75–84.68
Presence of dogs at sampling time				
No	6.3% (7/112)	1.77–10.73	35.3% (6/17)	12.58–58.01
Yes	17.8% (70/394)	13.99–21.54	71.4% (15/21)	52.11–90.75
Presence of dogs at the sampling site in the last two years				
No	5.1% (3/59)	0–10.69	25.0% (3/12)	0.50–49.50
Yes	16.6% (74/447)	13.11–20.00	69.2% (18/26)	51.49–86.97
Dogs’ access to the stable and/or pasture				
No	7.6% (9/119)	2.81–12.31	33.3% (6/18)	11.56–55.11
Yes	17.6% (9/387)	13.78–21.36	75.0% (15/20)	56.02–93.98
Presence of cats at sampling time				
No	14.2% (31/218)	9.58–18.86	51.7% (15/29)	33.54–69.91
Yes	16.0% (46/288)	11.74–20.20	66.7% (6/9)	35.87–97.47
Presence of cats at the sampling site in the last two years				
No	15.5% (28/181)	10.20–20.74	59.1% (13/22)	38.55–79.64
Yes	15.1% (49/325)	11.19–18.97	50.0% (8/16)	25.50–74.50
Cats’ access to the stable and/or pasture				
No	13.5% (15/111)	7.15–19.87	50.0% (9/18)	26.90–73.10
Yes	15.7% (62/395)	12.11–19.28	60.0% (12/20)	38.53–81.47
Animals purchase countries				
Italy	9.9% (12/121)	4.59–15.24	38.1% (8/21)	17.32–58.87
Italy and foreign countries	16.9% (65/385)	13.14–20.63	76.5% (13/17)	56.31–96.63
Europe	95.6% (65/68)	90.71–100.0	81.3% (13/16)	62.12–100.0
Americas	97.3% (36/37)	92.07–100.00	66.7% (2/3)	13.32–100.0
Oceania	100.0% (5/5)	100.00	100.0% (3/3)	100.0

### Statistical analysis

Regression analysis (Table 2) showed that *Sarcocystis* spp. seropositivity increased with age (OR = 1.076, 95% CI: 1.02–1.14, *p* = 0.0148). Therefore, age was included in all tested models as an effect-modifying explanatory variable. Risk factor analysis revealed a significant positive association between seropositivity to *Sarcocystis* spp. and *N. caninum* (OR = 3.047, 95% CI 1.26–7.35, *p* = 0.0132).

**Table 2:** Generalized linear mixed models showing potential risk factors for *Sarcocystis* spp. seropositivity in South American Camelids in Italy including into each of the models the variable “Sampling site ID” as a random effect variable and the variable “Age (in years)” as an effect modifying variable

Model (AIC)	Variable	Odds ratio (95% CI)	z-value	*p*-value
1 (429.5)	(Intercept)	0.103 (0.06–0.18)	-7.901	< 0.001
	Age (in years)	1.076 (1.02–1.14)	2.437	< 0.05
2 (426.0)	(Intercept)	0.361 (0.12–1.09)	-1.802	0.0715
	Age (in years)	1.073 (1.01–1.14)	2.302	< 0.05
	*Neospora caninum* ELISA competition percentage	0.988 (0.98–1.00)	-2.458	< 0.05
3 (426.0)	(Intercept)	0.098 (0.06–0.17)	-8.058	< 0.001
	Age (in years)	1.071 (1.01–1.14)	2.246	< 0.05
	*Neospora caninum* ELISA negative result (Ref.)	-	-	-
	*Neospora caninum* ELISA positive result	3.047 (1.26–7.35)	2.479	< 0.05
4 (424.1)	(Intercept)	0.063 (0.03–0.13)	-7.561	< 0.001
	Age (in years)	1.083(1.02–1.15)	2.615	< 0.01
	Number of dogs at the sampling site	1.528 (1.12–2.08)	2.705	< 0.01
5 (422.6)	(Intercept)	0.049 (0.02–0.11)	-7.344	< 0.001
	Age (in years)	1.084 (1.02–1.15)	2.621	< 0.01
	Dogs access to the stables and/or pasture – No (Ref.)	-	-	-
	Dogs access to the stables and/or pasture – Yes	3.191 (1.40–7.28)	2.764	< 0.01

*AIC* Akaike Information Index, *CI* Confidence Interval, *Ref*. reference

Specifically, an increase in ELISA competition percentage (S/N %) for *N. caninum*, indicating lower antibody titers, were inversely associated with *Sarcocystis* spp. seropositivity (OR = 0.988, 95% CI 0.98–1.00, *p* = 0.014). The access of dogs to stables and/or pasture was regarded as risk factor for *Sarcocystis* spp. seropositivity (OR = 3.191, 95% CI 1.40–7.28, *p* = 0.00572), particularly as the number of dogs increases (OR = 1.53, 95% CI: 1.12–2.08, *p* = 0.0068).

The final multivariable model (Table 3) confirmed a significant association between *Sarcocystis* spp. seropositivity and age (OR = 1.079, 95% CI 1.02–1.15, *p* = 0.01319), *N. caninum* seropositivity expressed as ELISA competition percentage (OR = 0.989, 95% CI 0.98–1.00, *p* = 0.03153), and access of dogs to stables and/or pasture (OR = 2.922, 95% CI 1.28–6.67, *p* = 0.01088).

**Table 3:** Final optimized generalized linear mixed multivariable model showing the identified risk factors for *Sarcocystis* spp. seropositivity in South American camelids in Italy including the variable “Sampling site ID” as a random effect variable and the variable “Age (in years)” as an effect modifying variable

Variable	Odds ratio Estimate (95% CI)	z-value	*p*-value
(Intercept)	0.152 (0.042–0.55)	-2.882	< 0.01
Age (in years)	1.079 (1.02–1.15)	2.479	< 0.05
*Neospora caninum* ELISA competition percentage	0.989 (0.98–1.00)	-2.150	< 0.05
Dogs access to the stables and/or pasture – No (Ref.)	-	-	-
Dogs access to the stables and/or pasture – Yes	2.922 (1.28–6.67)	2.547	< 0.05

*AIC *Akaike Information Index 420.3, *CI* Confidence Interval, *Ref.* reference

## Discussion

This study investigated for the first time the presence of *Sarcocystis* spp. antibodies in SAC sera in Italy, showing a seropositivity of 15.2%, corresponding to 14.4% in alpacas and 35.0% in llamas.

The prevalence obtained herein is lower than those found in alpacas in China (25.0% - Jiang et al. 2021), and Peru (100% - Fernandez et al. 2022), as well as in llamas in Argentina (95.8% - Moré et al.^2008^), however it is similar to those reported in llamas from Bolivia (34.1% - Rooney et al. 2014). In contrast, to the best of authors’ knowledge, no data from Europe are available for comparison with the results of this study, as it represents the first investigation of *Sarcocystis* spp. in SAC and the first serological survey conducted across the entire continent in these species.

The limited data on these protozoa in Europe are probably due to the lack of validated or commercially available serological diagnostic tools in live animals, making the diagnosis limited to direct methods or post-mortem findings (Decker Franco et al. 2018; Wieser et al. 2024; Neyra et al. 2025). Most investigations were carried out in South American countries where SAC meat is widely commercialized for human consumption, with Bolivia producing 16,554 tonnes in 2023 (14,664 from llamas and 1,890 from alpacas) (MDPRyA, 2024), and Peru producing 16,719 tonnes in 2016 (4,117 from llamas and 12,602 from alpacas) (MIDAGRI, 2019). In detail, in Bolivia and Argentina, *S. aucheniae* was detected through gross inspection of carcasses in 34.1% and 65.0% of llamas, respectively (Rooney et al. 2014; Decker Franco et al. 2018)d *masoni* was identified by histopathological examination of heart tissue in 100.0% of alpacas in Peru (Fernandez et al. 2022; Rodriguez et al. 2023). Seroprevalence of *Sarcocystis* spp. in llamas in Argentina was 95.8% by IFAT and 35.9% by ELISA (Moré et al. 2008a; Romero et al. 2017).

The lower prevalence found herein may be partly attributable to some limitations of the diagnostic method used, such as the use of *S. miescheriana* bradyzoites-based antigen. However, previous studies demonstrated that the detection of antibodies to *Sarcocystis* spp. is generally genus specific and cross-reactions among *Sarcocystis* species can occur (Moré et al. 2008a; Dubey et al. 2016; Saeed et al. 2018). Consequently, wild boar-derived bradyzoites may be used for the detection of anti-*Sarcocystis* spp. antibodies in SAC serum samples. However, a study comparing the serological detection of *Sarcocystis* spp. antibodies and the presence of sarcocysts in SAC muscles should be carried out for confirmation. Another possible reason might be the limited access of potential definitive hosts to SAC meat, likely preventing environmental contamination with oocysts/sporocysts.

With regards to the sex, (Romero et al. 2017) indicated male animals more likely to be infected, while (Rooney et al. 2014) reported females at higher risk due to possible changes in the immune system during pregnancy and parturition. However, this study found no significant association.

On the contrary, a significant association was found between the age of animals and risk of infection, with older age being a propensity factor for seropositivity. This result is in line with previous studies in camelids (Rooney et al. 2014; Romero et al. 2017; Wieser et al. 2024), suggesting that prolonged environmental exposure increases the risk of infection.

A recent metanalysis on *Sarcocystis* spp. in both OWC and NWC has shown that prevalence rates significantly vary between continents, with South America having the highest infection rates (Disfani et al. 2025). Unlike other coccidia, the maturation of *Sarcocystis* spp. sporocysts is not affected by weather conditions, altitude and/or pasture characteristics, because it occurs within the definitive host (Romero et al. 2017; Saeed et al. 2018). Accordingly, no statistically differences in prevalence was observed between Italian macro-areas, although the statistical power was low due to low number of samples tested from the South. Moreover, in southern Italy, the breeding of SACs is still poorly established, which may reduce opportunities for definitive hosts to become infected and, consequently, limit the circulation of the parasite. Therefore, the epidemiology of this protozoan infection is more likely affected by management practices. Romero et al. (2017) showed that llamas under good sanitation conditions (i.e., regular veterinary check-up, vaccinations, disease diagnosis and treatment) and absence of pastoral dogs have a lower likelihood of infection compared to those kept in rural context with informal care, lacking sanitary controls and co-living with pastoral dogs.

Although the life cycle of some *Sarcocystis* spp. has been well described in ruminants (Lindsay and Dubey 2020), knowledge of species involving SAC is still limited (Dubey et al. 2016; Wieser et al. 2024). Experimental infections have been conducted to identify the definitive host(s) of *Sarcocystis* species infecting SAC. In these studies dogs, cats, rats, and mice were fed with meat from infected SAC (alpaca, llama, and guanaco), and sporocysts were detected only in the faeces of dogs. These findings suggest that dogs are currently the only documented definitive hosts for both *S. aucheniae* (Gorman et al. 1984; Schnieder et al. 1984; Zacarías et al. 2013)d *masoni* (Wu et al. 2022). However, among these studies, only Wu et al. (2022) performed molecular analysis on the sporocysts for confirmation of *S. masoni*, while previous researchers relied only on microscopic detection of sporocyst in the faeces of the dogs after receiving *S. aucheniae* sarcocysts as conclusive evidence.

In this study, the results of the statistical analysis showed that the access of dogs to the stable and/or pasture significantly increases the risk for *Sarcocystis* spp. seropositivity (Tables 2 and 3), as well as the number of dogs at the sampling site increases (Table 2). However, no diagnostic tests were carried out on dogs sharing the same environment as the camelids included in this study; therefore, their role in the transmission of *Sarcocystis* spp. should be interpreted with caution. Consequently, in the lack of a specific treatment for sarcocystosis in intermediate hosts, prevention strategies should aim to break the perpetuation of the parasite cycle acting directly on the potential definitive hosts. This could be achieved by limiting dogs’ access to stable and pasture where SAC are kept (Rooney et al. 2014).

In this study seropositivity was recorded also in sampling site where cats were present (Table 1). Although felids are not typically known to shed *Sarcocystis* sporocysts infecting SAC, their role as potential definitive hosts cannot be totally ruled out. Therefore, it would be advisable to perform further studies employing molecular tools to better investigate the role of domestic and wild canids and felids, as well as other predators and scavengers in the transmission of *Sarcocystis* spp. in SAC. In South America, wild canids such as Andean foxes (*Lycalopex culpaeus*), and wild felids such as pumas (*Puma concolor*) represent the main SAC predators (Miranda de la Lama and Villarroel 2023) and, considering the frequent detection of *Sarcocystis* spp. in camelids in these areas, their potential role in the transmission of the parasite should be investigated.

Interestingly, 38.1% (8/21) of the positive sampling site reported keeping only Italian SAC (born at the same farm or purchased from other Italian farms). This finding, along with prior assumptions about the biological cycle of these protozoa, may suggest the local completion of the life cycle.

The same sera had previously been tested for the detection of other apicomplexan parasite (i.e., *N. caninum* and *T. gondii*) antibodies. The statistical analysis revealed a significant association between animals positive for *Sarcocystis* spp. and *N. caninum* (Table 2), while no association was observed with serological results on *T. gondii.* For this reason matching only the results of *Sarcocystis* spp. and *N. caninum*, 87.0% (67/77) of animals were seropositive exclusively for *Sarcocystis* spp., while 13.0% (10/77) were positive to both protozoa. The positive association with *N. caninum* was observed especially in case of animals with low S/N for *N. caninum* (Table 3; Table S1). Both protozoa are phylogenetically related and cross-reactions have been occasionally reported in cattle (García-Lunar et al. 2015). However, *N. caninum* positive samples were confirmed by immunoblot reducing the probability of false positive due to antigenic cross-reaction. Therefore, the co-infection between *Sarcocystis* spp. and *N. caninum* may be explained by a concurrent exposure, potentially related to the life cycle of these parasites and shared risk factors, such as the presence of dogs at the sampling site, which are definitive hosts for *N. caninum* and may also play a role in the environmental dissemination of *Sarcocystis* spp. sporocysts.

In Italy, SAC are generally kept under good management practices, with all owners reporting regular veterinary clinical examinations; however, the diagnosis of acute sarcocystosis can be difficult, as the disease may be subclinical or lack specific clinical signs (Saeed et al. 2018). This highlights the need for further research for developing improved serological methods for the in live diagnosis in SAC. Likewise, the increased implementation of necropsies, and histopathological examinations would be useful tools for further epidemiological studies.

## Conclusion

This study provides the first serological data on *Sarcocystis* spp. infection in alpacas and llamas in Italy, providing valuable data to the European epidemiological scenario. The results proved the circulation of *Sarcocystis* species affecting SAC in the country, and with a view to a future SAC meat supply chain, *Sarcocystis* infection may became a relevant concern for veterinary medicine. Furthermore, as the zoonotic potential has not yet been clarified, this aspect should be carefully monitored in the context of food safety. Therefore, proactive surveillance, standardization of diagnostic methods and integration of *Sarcocystis* infection into SAC health management protocols and necropsies are strongly recommended. Further research should focus on the molecular identification of the species circulating in Italy, as well as the role of carnivores in the life cycle of *Sarcocystis* spp. in SAC in order to limit the transmission of these protozoa.

## Supplementary Information

Supplementary Material 1.

Supplementary Material 2.

## Data Availability

The data generated and analyzed during this study are available from the corresponding author upon reasonable request.
