# Correction to: Serological investigation on Sarcocystis spp. infection and associated risk factors in South American camelids in Italy

**DOI:** 10.1007/s00436-026-08776-1

**Published:** 2026-10-08

**Authors:** Elisa Castaldo, Gastón Moré, Gereon Schares, Francesco Buono, Stefano Scarcelli, Giovanni Sgroi, Farwa Humak, Walter Basso, Vincenzo Veneziano

**Affiliations:** 1 Department of Veterinary Medicine and Animal Productions, University of Naples Federico II, Naples, Italy; 2 Institute of Parasitology, Vetsuisse-Faculty, University of Bern, Bern, Switzerland; 3 Federal Research Institute for Animal Health, Institute of Epidemiology, Friedrich-Loeffler Institut, Greifswald-Insel Riems, Germany; 4 Department of Animal Health, Experimental Zooprohylactic Institute of southern Italy, Portici, Italy; 5 Department of Sciences and Technologies, University of Sannio, Benevento, Italy

**Correction to: Parasitology Research**

**https://doi.org/10.1007/s00436-026-08655-9**

The authors regret that the text that reads as:

Of the study animals, 64.2% (325/506) were females, 31.6% (160/325) males, and 4.2% (21/506) castrated.

under the **Materials and methods section** (Study animals and sampling sites, page 2) of the original published article is incorrect. The correct text should read as:

Of the study animals, 64.2% (325/506) were females, 31.6% (160/506) males, and 4.2% (21/506) castrated.

The original article has been corrected.

