# Supplementary material for: Serological investigation on Sarcocystis spp. infection and associated risk factors in South American camelids in Italy
Source: Parasitol Res. 2026 Mar 4;125(1):35. doi: 10.1007/s00436-026-08655-9 (PMC12963157; doi:10.1007/s00436-026-08655-9)
Supplement: Supplementary file 1 — Supplementary Material 1. [file 436_2026_8655_MOESM1_ESM.docx]

**Supplementary Table S1.** Seropositivity for *Sarcocystis* spp. and *N. caninum*

| **Animal ID** | **Species** |  | ***Sarcocystis* spp.**  **IFAT** | |  | ***Neospora caninum***  **Competitive ELISA** | |
| --- | --- | --- | --- | --- | --- | --- | --- |
|  |  |  | **Result**  **(+/-)** | **Titer** |  | **Result**  **(+/-)** | **S/N value**  **(%)** |
| 10 | Alpaca |  | - | 1:25 |  | + | 7.34 |
| 17 | Alpaca |  | + | 1:25 |  | - | 99.66 |
| 34 | Alpaca |  | + | 1:25 |  | - | 95.67 |
| 35 | Alpaca |  | + | 1:25 |  | - | 96.35 |
| 37 | Alpaca |  | + | 1:25 |  | - | 110.95 |
| 39 | Alpaca |  | + | 1:25 |  | - | 102.13 |
| 40 | Alpaca |  | + | 1:25 |  | - | 95.71 |
| 44 | Alpaca |  | + | 1:25 |  | - | 112.82 |
| 45 | Alpaca |  | + | 1:25 |  | - | 110.43 |
| 47 | Alpaca |  | + | 1:25 |  | - | 108.63 |
| 52 | Alpaca |  | + | 1:25 |  | - | 114.94 |
| 56 | Alpaca |  | + | 1:25 |  | - | 101.46 |
| 58 | Alpaca |  | + | 1:25 |  | - | 99.70 |
| 59 | Alpaca |  | + | 1:25 |  | - | 100.80 |
| 61 | Alpaca |  | + | 1:25  1:50 |  | - | 104.62 |
| 62 | Alpaca |  | + | 1:25 |  | - | 102.80 |
| 69 | Alpaca |  | + | 1:25 |  | - | 108.32 |
| 78 | Alpaca |  | + | 1:25 |  | - | 100.70 |
| 83 | Alpaca |  | + | 1:25 |  | + | 20.57 |
| 86 | Alpaca |  | - | 1:25 |  | + | 23.22 |
| 87 | Alpaca |  | + | 1:25 |  | - | 107.35 |
| 92 | Alpaca |  | + | 1:25 |  | - | 96.40 |
| 94 | Alpaca |  | + | 1:25 |  | - | 103.74 |
| 95 | Alpaca |  | + | 1:25 |  | - | 104.65 |
| 101 | Alpaca |  | + | 1:25 |  | + | 30.50 |
| 103 | Alpaca |  | + | 1:25 |  | + | 17.91 |
| 105 | Alpaca |  | + | 1:25 |  | - | 94.16 |
| 109 | Alpaca |  | + | 1:25 |  | - | 95.07 |
| 112 | Alpaca |  | - | 1:25 |  | + | 18.82 |
| 138 | Alpaca |  | + | 1:25 |  | - | 94.09 |
| 149 | Alpaca |  | + | 1:25 |  | - | 100.93 |
| 150 | Alpaca |  | + | 1:25 |  | - | 113.01 |
| 153 | Alpaca |  | + | 1:25  1:50 |  | - | 107.60 |
| 158 | Alpaca |  | + | 1:25 |  | - | 99.07 |
| 161 | Alpaca |  | - | 1:25 |  | + | 35.28 |
| 162 | Alpaca |  | + | 1:25 |  | - | 120.78 |
| 163 | Alpaca |  | + | 1:25 |  | - | 97.43 |
| 167 | Llama |  | + | 1:25 |  | - | 101.89 |
| 168 | Llama |  | + | 1:25 |  | - | 120.67 |
| 178 | Alpaca |  | + | 1:25 |  | - | 110.65 |
| 180 | Alpaca |  | + | 1:25 |  | + | 16.73 |
| 181 | Alpaca |  | - | 1:25 |  | + | 16.12 |
| 183 | Alpaca |  | + | 1:25  1:50 |  | + | 24.39 |
| 186 | Alpaca |  | - | 1:25 |  | + | 36.81 |
| 187 | Alpaca |  | - | 1:25 |  | + | 14.94 |
| 189 | Alpaca |  | - | 1:25 |  | + | 17.07 |
| 192 | Alpaca |  | + | 1:25 |  | + | 8.46 |
| 193 | Alpaca |  | + | 1:25 |  | + | 10.10 |
| 194 | Alpaca |  | + | 1:25 |  | - | 109.05 |
| 198 | Alpaca |  | - | 1:25 |  | + | 48.60 |
| 202 | Alpaca |  | + | 1:25 |  | - | 110.31 |
| 204 | Alpaca |  | + | 1:25 |  | - | 114.38 |
| 207 | Alpaca |  | + | 1:25 |  | - | 98.11 |
| 208 | Alpaca |  | + | 1:25  1:50 |  | - | 95.45 |
| 219 | Alpaca |  | + | 1:25 |  | - | 104.63 |
| 248 | Alpaca |  | + | 1:25 |  | - | 92.80 |
| 250 | Alpaca |  | - | 1:25 |  | + | 7.85 |
| 251 | Alpaca |  | + | 1:25 |  | - | 98.64 |
| 269 | Alpaca |  | - | 1:25 |  | + | 22.96 |
| 274 | Alpaca |  | + | 1:25 |  | - | 120.80 |
| 275 | Llama |  | + | 1:25  1:50  1:100 |  | - | 111.44 |
| 276 | Llama |  | + | 1:25 |  | - | 125.96 |
| 277 | Llama |  | + | 1:25 |  | - | 111.22 |
| 279 | Alpaca |  | - | 1:25 |  | + | 7.96 |
| 286 | Alpaca |  | - | 1:25 |  | + | 48.79 |
| 291 | Alpaca |  | + | 1:25 |  | - | 104.11 |
| 292 | Alpaca |  | + | 1:25 |  | - | 118.93 |
| 295 | Alpaca |  | - | 1:25 |  | + | 11.69 |
| 301 | Alpaca |  | - | 1:25 |  | + | 5.68 |
| 312 | Alpaca |  | + | 1:25 |  | - | 111.31 |
| 320 | Alpaca |  | + | 1:25 |  | - | 115.63 |
| 326 | Alpaca |  | + | 1:25 |  | - | 109.89 |
| 334 | Alpaca |  | + | 1:25 |  | - | 101.61 |
| 339 | Alpaca |  | - | 1:25 |  | + | 11.14 |
| 360 | Alpaca |  | - | 1:25 |  | + | 43.25 |
| 365 | Alpaca |  | + | 1:25 |  | - | 108.30 |
| 367 | Alpaca |  | - | 1:25 |  | + | 15.71 |
| 368 | Alpaca |  | + | 1:25 |  | + | 19.65 |
| 371 | Alpaca |  | + | 1:25 |  | - | 104.83 |
| 377 | Alpaca |  | + | 1:25  1:50 |  | - | 106.86 |
| 390 | Alpaca |  | + | 1:25 |  | - | 98.85 |
| 391 | Alpaca |  | + | 1:25 |  | - | 92.48 |
| 392 | Alpaca |  | + | 1:25 |  | - | 98.81 |
| 396 | Alpaca |  | + | 1:25 |  | - | 116.55 |
| 402 | Alpaca |  | + | 1:25 |  | - | 102.72 |
| 406 | Alpaca |  | + | 1:25 |  | - | 105.72 |
| 416 | Alpaca |  | + | 1:25  1:50 |  | - | 115.37 |
| 418 | Alpaca |  | + | 1:25 |  | - | 113.23 |
| 419 | Llama |  | + | 1:25 |  | + | 9.22 |
| 442 | Alpaca |  | + | 1:25 |  | - | 93.71 |
| 476 | Llama |  | + | 1:25 |  | - | 97.46 |
| 479 | Llama |  | - | 1:25 |  | + | 49.01 |
| 482 | Alpaca |  | + | 1:25 |  | - | 92.57 |
| 484 | Alpaca |  | + | 1:25 |  | + | 8.57 |
| 491 | Alpaca |  | + | 1:25 |  | - | 90.98 |
| 505 | Alpaca |  | + | 1:25 |  | - | 94.66 |

+ = positive, - = negative, S/N = competition percentage
