# Supplementary material for: Serological investigation on Sarcocystis spp. infection and associated risk factors in South American camelids in Italy
Source: Parasitol Res. 2026 Mar 4;125(1):35. doi: 10.1007/s00436-026-08655-9 (PMC12963157; doi:10.1007/s00436-026-08655-9)
Supplement: Supplementary file 2 — Supplementary Material 2. [file 436_2026_8655_MOESM2_ESM.pdf]

Supplementary material S1. Questionnaire used to obtain data on south American Camelids owners, farm veterinarian, farm structure and management, animal health management and clinical history

UNIVERSITÀ DEGLI STUDI DI NAPOLI FEDERICO II

Dipartimento Medicina Veterinaria Produzioni Animali

UNINA

INFORMED CONSENT TO PARTICIPATE IN THE STUDY

The undersigned_______________________________, residing in _____________________ Province (____), address _______________________________, owner of the alpaca/llama with name______________, sex____, years_________, microchip number ___________________, declares to voluntarily participate in the aforementioned study, authorizing the sampling of faeces, blood and serological investigations as routine health control practices.

Furthermore, the undersigned declares to have been informed that the personal data provided will be used only for the purposes of the research activity of the Department of Veterinary Medicine and Animal Production of the University of Naples Federico II and may not be disclosed to third parties pursuant to of Legislative Decree 1096/2003.

Location and date ________________________

Declarant's signature ______________________________

QUESTIONNAIRE SURVEY ON ALPACA AND LLAMA PARASITES

Date: ____________________

1. FIRST SECTION – OWNER DATAs

**1.1 Name and surname:**	
**1.2 E-mail address:**	
**1.3 Mobile number:**	
**1.4 Farm name:**	
**1.5 Farm city and province:**	
**1.6 Farm address:**	
**1.7 Study title:**	☐ Elementary education<br>☐ Middle school<br>☐ High School<br>☐ Degree<br>☐ Specialization/Master<br>☐ Doctoral
**1.8 Employment:**	Specify: ____________________________________

2. SECOND SECTION – VETERINARY DATAs

**2.1 Name and surname:**	
**2.2 E-mail address:**	
**2.3 Mobile number:**	

FARM DATA

3. THIRD SECTION – FACILITIES OF THE FARMS

**3.1 Where is your farm located in?**	☐ Northern Italy (Emilia Romagna, Friuli-Venezia Giulia, Liguria, Lombardia, Piemonte, Trentino-Alto Adige, Valle d'Aosta, Veneto)<br>☐ Central Italy (Abruzzo, Lazio, Marche, Toscana, Umbria)<br>☐ Southern Italy (Basilicata, Calabria, Campania, Molise, Puglia)<br>☐ Islands (Sardegna, Sicilia)
**3.2 When was your farm founded?**	☐ Specify the year: ________________
**3.3 How do you receive information about camelids management?**	☐ Books<br>☐ Seminars<br>☐ Camelid breeders<br>☐ Livestock animals breeders<br>☐ Web research/web forum

	☐ Scientific journals<br>☐ Magazine<br>☐ Veterinarian<br>☐ Other (specify: ____________________)
**3.4 How many people are involved in taking care of your camelids?**	☐ 1<br>☐ 2<br>☐ 3<br>☐ 4<br>☐ 5<br>☐ >5
**3.5 What is primary function of your alpacas/llamas?**	☐ Hobby<br>☐ Wool production<br>☐ Trekking<br>☐ Pet-therapy<br>☐ Meat<br>☐ Breeder<br>☐ Other (specify: ____________________________________)
**3.6 How many alpacas/llamas are in your farm?**	☐ Alpacas (number: ________)<br>☐ Llamas (number: _________)<br>☐ Other camelids (specify: ____________)
**3.7 Herd size**	☐ Small (1-20)<br>☐ Medium (> 20 to ≤ 50)<br>☐ Large (>50)
**3.8 What breed of alpacas do you keep?**	☐ Suri (number: ________)<br>☐ Huacaya (number: ________)<br>☐ Crossbreed (specify: _____________________ number: ________)
**3.9 What is the origin of your animals?**	☐ Nationally<br>☐ Foreign (Country: ______________________________)
**3.10 Do you perform a quarantine period when a new animal is introduced?**	☐ No<br>☐ Yes<br>☐ < 1 week<br>☐ 1 week<br>☐ 2 weeks<br>☐ 3 weeks<br>☐ 4 weeks<br>☐ 5 weeks<br>☐ 6 weeks<br>☐ > 6 weeks

**3.11 How many categories of alapcas/llamas do you keep in your farm?**	☐ Cria (< 6 months)<br>- Total number: _________<br>- intact males: ___________<br>- females_________________<br>- castrated males _________<br>☐ Weaner (6-12 months)<br>- Total number: _________<br>- intact males: ___________<br>- females_________________<br>- castrated males _________<br>☐ Tuis (1-2 years)<br>- Total number: _________<br>- intact males: ___________<br>- females_________________<br>- castrated males _________<br>☐ Adults (> 2 years)<br>- Total number: _________<br>- intact males: ___________<br>- females_________________<br>- castrated males _________
**3.12 What is the average weaning age of crias?**	☐ 3 months<br>☐ 4 months<br>☐ 5 months<br>☐ 6 months<br>☐ Other: ______________________________
**3.13 Do you keep lactating females and weaned crias separately?**	☐ No<br>☐ Yes
**3.14 Do you keep your animals in group?**	☐ No<br>☐ Yes
**3.15 If yes, how many animals each group is made up?**	☐ <10 animals<br>☐ 10-20 animals<br>☐ >20 animals<br>☐ Other: __________________________
**3.16 What animal(s) is/are present in your farm?**	☐ Cats (number: _________)<br>☐ Dogs (number: _________)<br>☐ Horses (number: _________)<br>☐ Donkeys (number: _________)<br>☐ Buffaloes (number: _________)<br>☐ Sheep (number: _________)<br>☐ Goat (number: _________)<br>☐ Pigs (number: _________)<br>☐ Other animal species (specify: __________number:_________)<br>☐ No other animals
**3.17 Do alpacas/llamas**	☐ No<br>☐ Yes

**on your farm share the same environments with other livestock species? If yes, specify**	☐ Box (specify the livestock specie: ________________)<br>☐ Paddock (specify the livestock specie: ________________)<br>☐ Pasture (specify the livestock specie: ________________)
**3.18 Contact with wild animals**	☐ No<br>☐ Yes (specify: __________)
**3.19 Are wild animals netting present in you farm?**	☐ No<br>☐ Yes
**3.20 How are alpacas/llamas housed?**	☐ Stable and pasture<br>☐ Pasture/paddock with shelter<br>☐ Stable with paddock<br>☐ Pasture<br>☐ Stable<br>☐ Other (specify: __________________)
**3.21 What is the size of the stable?**	☐ Number of animals: _____________ m^2:________________<br>☐ Number of animals: _____________ m^2:________________<br>☐ Number of animals: _____________ m^2:________________
**3.22 What type of bedding is present?**	☐ Concrete<br>☐ Hay<br>☐ Straw<br>☐ Rubber mats<br>☐ Wood shavings<br>☐ Wooden floor<br>☐ Sand<br>☐ Ground<br>☐ Other (specify: _______________________________________)
**3.23 Do alpacas/llamas have access to pasture?**	☐ No<br>☐ Yes<br>Jan. ☐ Feb. ☐ Mar. ☐ Apr. ☐ May ☐ Jun. ☐ Jul. ☐ Aug. ☐ Sep. ☐ Oct. ☐ Nov. ☐ Dec. ☐
**3.24 Do you perform pasture rotation system?**	☐ No<br>☐ Yes
**3.25 Do you do pasture cleaning? If yes, how often do you do it?**	☐ No<br>☐ Yes<br>☐ Daily<br>☐ Weekly<br>☐ Monthly<br>☐ Once a year<br>☐ Twice a year<br>☐ Other _______________________________________
**3.26 Do you do paddock cleaning?**	☐ No<br>☐ Yes<br>☐ Daily<br>☐ Weekly

**If yes, how often do you do it?**	☐ Monthly<br>☐ Once a year<br>☐ Twice a year<br>☐ Other: ______________________
**3.27 Do you do box cleaning? If yes, how often do you do it?**	☐ No<br>☐ Yes<br>☐ Daily<br>☐ Weekly<br>☐ Monthly<br>☐ Once a year<br>☐ Twice a year<br>☐ Other: ______________________
**1.28 Do you shear the animals? If yes, In which months?**	☐ No<br>☐ Yes<br>Jan. ☐ Feb. ☐ Mar. ☐ Apr. ☐ May ☐ Jun. ☐ Jul. ☐ Aug. ☐ Sep. ☐ Oct. ☐ Nov. ☐ Dec. ☐
**3.29 Do you cut toenails? If yes, how often do you do it?**	☐ No<br>☐ Yes<br>☐ If needed<br>☐ Once a year<br>☐ Twice a year<br>☐ More than twice a year
**3.30 Do you trim teeth? If yes, how often do you do it?**	☐ No<br>☐ Yes<br>☐ If needed<br>☐ Once a year<br>☐ Twice a year<br>☐ More than twice a year
**3.31 Are there any water courses near the farm? If yes, specify the type**	☐ No<br>☐ Yes<br>☐ Stream<br>☐ River<br>☐ Lake<br>☐ Swamp<br>☐ Irrigation canals
**3.32 What is the water source of animals?**	☐ Individual drinking trough<br>☐ Collective water tanks<br>☐ Natural water courses (i.e. streams, rivers, lakes)
**3.33 What is the main feed?**	☐ Pasture grass<br>☐ Hay<br>☐ Haylage<br>☐ Silage<br>☐ Straw<br>☐ Other: ______________________
**3.34 Do you add other**	☐ No<br>☐ Mineral feed<br>☐ Concentrate

**components to the main diet?**	☐ Vegetables<br>☐ Fruit<br>☐ Vitaminic supplement<br>☐ Other: ______________________________
**3.35 Presence of own cats on the farm in the last 2 years. If yes, specify**	☐ No<br>☐ Yes<br>☐ < 5 cats<br>☐ 5-10 cats<br>☐ > 10 cats
**3.36 Have there been kittens (<6 months) on the farm in the last 2 years?**	☐ No<br>☐ Yes<br>☐ I don't know
**3.37 Did the cats have access to the barn and/or pasture?**	☐ No<br>☐ Yes<br>☐ I don't know
**3.38 Can stray cats or neighbour's cats have access to the stable and/or pasture of your farm?**	☐ No<br>☐ Yes<br>☐ I don't know
**3.39 Presence of own dogs on the farm in the last 2 years. If yes, specify**	☐ No<br>☐ Yes<br>☐ < 5 dogs<br>☐ 5-10 dogs<br>☐ > 10 dogs
**3.40 Have there been puppies (<6 months) on the farm in the last 2 years?**	☐ No<br>☐ Yes<br>☐ I don't know
**3.41 Did the dogs have access to the barn and/or pasture?**	☐ No<br>☐ Yes<br>☐ I don't know
**3.42 Can stray dogs or neighbour's dogs have access to the stable and/or pasture of your farm?**	☐ No<br>☐ Yes<br>☐ I don't know

4. FOURTH SECTION - SANITARY PROCEDURES

**4.1 Do you follow a vaccination program?**	☐ No<br>☐ Yes<br>☐ Clostridial infections<br>☐ Tetanus<br>☐ Chlamydial abortion<br>☐ Bluetongue disease<br>☐ Rabies<br>☐ Other (specify: ______________________)
**4.2 In which month(s)?**	Jan. ☐ Feb. ☐ Mar. ☐ Apr. ☐ May ☐ Jun. ☐ Jul. ☐ Aug. ☐ Sep. ☐ Oct. ☐ Nov. ☐ Dec. ☐
**4.3 Do you perform treatment against ectoparasites?**	☐ No<br>☐ Yes
**4.4 What is your deworming routines in the farm?**	☐ Individually<br>☐ Category (cria, weaner, tui, adults)<br>☐ All animals simultaneously<br>☐ Other: ____
**4.5 What is the route of administration?**	☐ Injection<br>☐ Oral administration<br>☐ Topical application<br>☐ Pour-on<br>☐ Spot-on<br>☐ Other: ____
**4.6 How often do you perform treatment against ectoparasites?**	☐ Less than once a year<br>☐ Once a year<br>☐ Twice a year<br>☐ Three times a year<br>☐ Four times a year<br>☐ Following a diagnosis<br>☐ Other: ____
**4.7 In which month(s) do you usually perform treatment against ectoparasites?**	Jan. ☐ Feb. ☐ Mar. ☐ Apr. ☐ May ☐ Jun. ☐ Jul. ☐ Aug. ☐ Sep. ☐ Oct. ☐ Nov. ☐ Dec. ☐
**4.8 What drugs against ectoparasites do you use in the farm?**	☐ Ivermectin<br>☐ Moxidectin<br>☐ Doramectin<br>☐ Eprinomectin<br>☐ Pyrethroids<br>☐ Organophosphate<br>☐ Phytosanitary<br>☐ Other: __________________________________
**4.9 Do you perform treatment against endoparasites?**	☐ No<br>☐ Yes

**4.10 What is your deworming routines in the farm?**	☐ Individually<br>☐ Category (cria, weaner, tui, adults)<br>☐ All animals simultaneously<br>☐ Other: ______________________
**4.11 How do you decide when to deworm an animal?**	☐ Strategic treatment (performed at regular intervals of time)<br>☐ Below the veterinarian tip<br>☐ If clinical signs are observed<br>☐ If parasite/parasitic elements are found in faeces<br>☐ Low production<br>☐ Other: ______________________
**4.12 What is the route of administration?**	☐ Injection<br>☐ Oral administration<br>☐ Topical application<br>☐ Pour-on<br>☐ Spot-on<br>☐ Other: ______________________
**4.13 How often do you perform treatment against endoparasites?**	☐ Less than once a year<br>☐ Once a year<br>☐ Twice a year<br>☐ Three times a year<br>☐ Four times a year<br>☐ Following parasitological faecal analysis<br>☐ Other: ______________________
**4.14 In which month(s) do you usually perform treatment against endoparasites?**	Jan. ☐ Feb. ☐ Mar. ☐ Apr. ☐ May ☐ Jun. ☐ Jul. ☐ Aug. ☐ Sep. ☐ Oct. ☐ Nov. ☐ Dec. ☐
**4.15 What type of deworming drugs used in the farm?**	☐ Ivermectin (Ecomectin, Ivertin, Ivomec, Maximec, Noromectin, Oramec, Tolomec, Vectimax, Vectimec, Virbamec)<br>☐ Moxidectin (Cydectin)<br>☐ Doramectin (Dectomax)<br>☐ Fenbendazole (Panacur; Vermovin)<br>☐ Albendazole (Alphalben; Sverminator; Zodalben)<br>☐ Pyrantel<br>☐ Praziquantel (Detenase; Neomansonil)<br>☐ Toltrazuril (Baycox multi; Tolracol; Toltranil)<br>☐ Monepantel (Zolvix)<br>☐ Levamisole (Pamizole L)<br>☐ Closantel/Oxifendazolo (Oxydrench)<br>☐ Closantel/Mebendazolo (Seponver Plus)<br>☐ Other: ______________________
**4.16 Which dose do you use?**	☐ Dose used for sheep<br>☐ Dose used for sheep x 1.5<br>☐ Dose used for sheep x2<br>☐ Dose used for sheep x4<br>☐ Other: ______________________
**4.17 Who performs the treatment?**	☐ Yourself<br>☐ Veterinarian<br>☐ Llama/alpaca breeder

	☐ Other: ___________________
**4.18 Do you move alpacas/llamas to clean pasture after deworming?**	☐ No<br>☐ Yes
**4.19 Do you calculate the weight of the alpacas/llamas? If yes specify**	☐ No<br>☐ Yes<br>☐ Visual estimation<br>☐ According to age<br>☐ By weighing scale<br>☐ Other: ___________________
**4.20 Do you evaluate BCS? If yes specify**	☐ No<br>☐ Yes<br>☐ Visual estimation<br>☐ Palpation of the lumbar region<br>☐ Other: ___________
**4.21 Do you deworm new alpaca/llama before introducing them in your herds?**	☐ No<br>☐ Yes
**4.22 How often do you perform parasitological faecal analysis?**	☐ Less than once a year<br>☐ Once a year<br>☐ Twice a year<br>☐ Three times a year<br>☐ Four times a year<br>☐ Other: ___________________
**4.23 Who performs the sampling?**	☐ Yourself<br>☐ Veterinarian<br>☐ Llama/alpaca breeder<br>☐ Other: ___________________
**4.24 Do you monitor the success of the treatment by coprological examination**	☐ No<br>☐ Yes
**2.25 Is there any dewormer which is resistant on your farm?**	☐ No<br>☐ Yes (specify: ___________)<br>☐ I don't know
**4.26 Did you observe the presence of parasite/parasitic elements in alpaca/llama feces?**	☐ No<br>☐ Yes
**4.27 Which age group(s) of alpacas/llamas**	☐ Cria (< 6 months)<br>☐ Weaners (6-12 months)<br>☐ Tuis (1-2 years)

**is/are mostly affected by endoparasites in your experience?**	☐ Adults (> 2 years)<br>☐ I don't know
**4.28 Which age group(s) of alpacas/llamas is/are mostly affected by ectoparasites in your experience?**	☐ Cria (< 6 months)<br>☐ Weaners (6-12 months)<br>☐ Tuis (1-2 years)<br>☐ Adults (> 2 years)<br>☐ I don't know
**4.29 In which month(s) do you usually observe problems associated with endoparasites in alpacas/llamas?**	Jan. ☐ Feb. ☐ Mar. ☐ Apr. ☐ May ☐ Jun. ☐ Jul. ☐ Aug. ☐ Sep. ☐ Oct. ☐ Nov. ☐ Dec. ☐
**4.30 In which month(s) do you usually observe problems associated with ectoparasites in alpacas/llamas?**	Jan. ☐ Feb. ☐ Mar. ☐ Apr. ☐ May ☐ Jun. ☐ Jul. ☐ Aug. ☐ Sep. ☐ Oct. ☐ Nov. ☐ Dec. ☐
**4.31 Which disease(s) occur in your farm about gastrointestinal system?**	☐ None<br>☐ Gastrointestinal parasites<br>☐ Diarrhoea<br>☐ Anorexia<br>☐ Colic<br>☐ Inappetence<br>☐ Dental problems<br>☐ Other: ______________________
**4.32 Which dermatological disease(s) occur in your farm?**	☐ Noone<br>☐ Ectoparasites<br>☐ Abscess<br>☐ Mycosis<br>☐ Lesions<br>☐ Dermatitis<br>☐ Other: ______________________
**4.33 Which disease(s) occur in your farm about reproductive system?**	☐ None<br>☐ Abortions<br>☐ Stillbirths<br>☐ Difficult births<br>☐ Stunted growth<br>☐ Malformation in crias<br>☐ Lack of milk in females<br>☐ Fertility disorder<br>☐ Other: ____________________________
**4.34 Which disease(s) occur in your farm about**	☐ None<br>☐ Anemia<br>☐ Hearth murmurs

**cardiovascular system?**	☐ Other: ____________________
**4.35 Which disease(s) occur in your farm about respiratory system?**	☐ None<br>☐ Cough<br>☐ Tachypnoea<br>☐ Nasal discharge<br>☐ Wheezing<br>☐ Pneumonia<br>☐ Bronchitis<br>☐ Other: ____________________
**4.36 Which disease(s) occur in your farm about skeletal muscle system?**	☐ None<br>☐ Fractures<br>☐ Lameness<br>☐ Exposed fractures<br>☐ Other: ____________________
